# Supplementary material for: Discriminating North American Swine Influenza Viruses with a Portable, One-Step, Triplex Real-Time RT-PCR Assay, and Portable Sequencing
Source: Viruses. 2024 Sep 30;16(10):1557. doi: 10.3390/v16101557 (PMC11512246; doi:10.3390/v16101557)
Supplement: Supplementary file 1 [file viruses-16-01557-s001.zip › viruses-3227881-supplementary.pdf]

Supplementary Materials

Figure S1. The sequence alignments of swine influenza triplex real-time RT-PCR assay with reference viruses

Figure S1a. The sequence alignment of sH1 probe with reference viruses

```
                                          Forward primer               Probe complimentary              Reverse complimentary
                                           920        930               950        960        970             1030      1040
                                        ....|....|....|....|...    .|....|....|....|....|....|....|    ....|....|....|....|...
sH1 Primer Probe                        CTTCCTTTCCAGAAYGTACAYCC~~~CAATAGGAGARTGTCCAAARTATGTCAGGAGT~~~CAATCCAGAGGTTTGTTTGGAGC
         ┌ A/Michigan/383/2018          .....C.................    ................................    .......................
1B.2.1 { A/California/62/2018          .....C.................    ................................    .......................
         │ A/Ohio/24/2018               .....C.................    ................................    .......................
         └ A/Ohio/35/2017               .......................    ................................    .......................
         ┌ A/Minnesota/70/2016          .......................    ................................    ....................G..
1B.2.2 { A/Wisconsin/71/2016           .......................    ................................    ....................G..
         │ A/Iowa/32/2016               .......................    ................................    ....................G..
         └ A/Minnesota/19/2011          .......................    ................................    ....................G..
Human H1 { A/Brisbane/59/2007           .......................    ................................    .......................
```

Figure S1b. The sequence alignment of H3v_2010 probe with reference viruses

```
                                                  Forward            Probe complimentary     Reverse complimentary
                                               910        920      1005       1015      1030      1040      1050
                                             ....|....|....|....|.    .|....|....|....|    |....|....|....|....|..
H3v 2010 Primer Probe                        AAGCATTCCMAATGACAAACC~~~GAATGAGAAATATACCA~~~ACTAGRGGCATATTYGGCGCAAT
      ┌ 2010.1      ┌A/Ohio/13/2017          .....................    .................    .......................
      │             └A/Ohio/28/2016          .....................    .................    .......................
H3v { Cluster       ┌A/Manitoba/03/2021      .....................    .....C.G...G.G..G    ........A........T.....
      │  IV         └A/West_Virginia/06/2011 ......T..............    .....C.G...G.....    .......................
      └ Cluster I {A/Hawaii/28/2020          .....................    .G...C.G...G.....    .......................
               ┌A/Hong Kong/2671/2019        .....................    .....C.....G.....    .................T.....
               │A/Kansas/14/2017             .....................    .....C.....G.....    .......................
Seasonal H3 {  A/Hong_Kong/4801/2014         .....................    .....C.....G.....    .......................
               │A/Texas/50/2012              .....................    .....C.G...G.....    .......................
               │A/Perth/16/2009              .....................    .G...C.....G.....    .......................
               └A/Wisconsin/67/2005          .....................    .G...C.....G.....    .......................
```

Figure S1c. The sequence alignment of cH1 probe with reference viruses

```
                                       Forward Primer              Probe complimentary         Reverse complimentary
                                        920       930       940          960       970         1010      1020      1030
                                    ....|....|....|....|....|    ...|....|....|....|....|..    ..|....|....|....|....|.
Classical H1 Primer Probe           GTGCTATAAACACCAGCCTYCCATT~~~ATCCAGTCACAATTGGgGAATGTCCA~~~GCYACAGGATTAAGGAATATCCCG
Classical H1 Forward_2              ....CC.............C.....
1A.1┌ A/Ohio/24/2017                ....CC...................    ................A.........    ..............A........A
    └ A/Minnesota/45/2016           ....CC...................    ................A.........    ..............A........A
1A.2┌ A/Minnesota/10/2009           .........................    ................A.........    ..............A.........
    └ A/Texas/14/2008               .........................    ................A.........    ..............A.........
    ┌ A/Wisconsin/03/2021           .........................    ................A.........    ........T..............A
1A.3│ A/Ohio/09/2015                .......T.................    ......................C...    ...........G.....C......
    │1A.3.3.2/ ┌ A/Michigan/45/2015 .........................    ....GA..........AA........    ...........G......G.....
    └H1N1pdm09 └ A/California/7/2009.........................    ....GA..........AA........    ...........G......G.T...
```

g(actually c)at nucleotide 968 was introduced into probe to discriminate pdmH1 viruses

Figure S2. Flow diagram of in-field testing

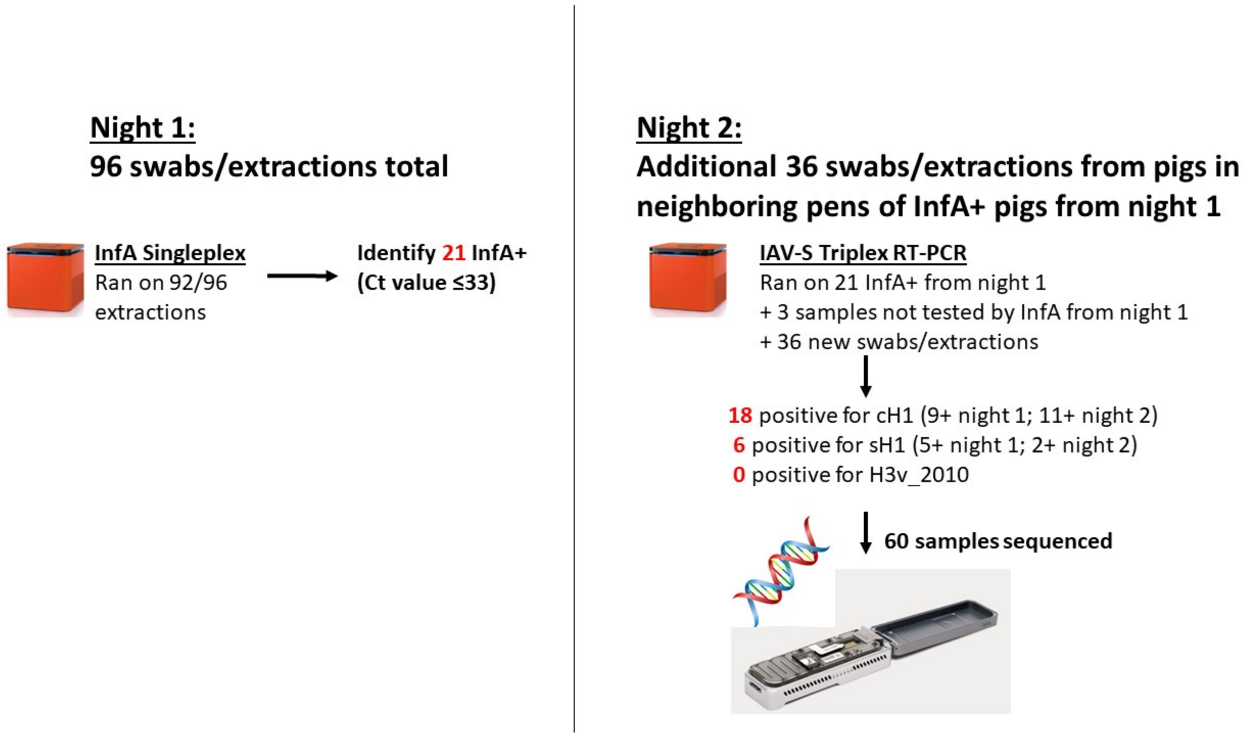

Table S1. Assay limit of detection with swine influenza H1 and swine H3 2010.1 clade viruses with original design.

Influenza Variant viruses (EID_{50}/mL)	InfA (Fam)	IAV-S_Triplex		
		sH1(Fam)	H3v_2010(Hex)	cH1(Cy5)
A/Ohio/35/2017(H1N2)v 1B.2.1				
$10^{5.9}$	17.75±0.08	20.64±0.10	-	-
$10^{3.9}$	30.79±0.30	31.22±0.25	-	-
$10^{2.9}$	36.61±0.68	-	-	-
$10^{1.9}$	-	-	-	-
A/Ohio/27/2016(H3N2)v 2010.1				
$10^{5.1}$	17.4±0.05	-	24.69±0.11	-
$10^{4.1}$	22.64±0.04	-	28.74±0.06	-
$10^{3.1}$	29.92±0.75	-	-	-
$10^{2.1}$	32.6±1.31	-	-	-
$10^{1.1}$	-	-	-	-
A/Ohio/24/2017(H1N2)v 1A.1.1				
$10^{5.3}$	17.40±0.27	-	-	22.85±0.05
$10^{3.3}$	28.06±0.44	-	-	30.41±1.62
$10^{2.3}$	32.75±0.32	-	-	35.3±3.5
$10^{1.3}$	35.68±0.41	-	-	-

Table S2. Test of performance of Akonni extraction and IAV-S Triplex on QuantaBio "Q" qPCR instrument (N=1).

Swine Influenza A Virus (RNA serial dilutions)	Ct value of IAV-S rRT-PCR		
	sH1(FAM)	H3v_2010(Hex)	cH1(Cy5)
A/Michigan/383/2018(H1N2)v 1B.2.1			
neat	17.41	-	-
10^{-1}	20.67	-	-
10^{-2}	24.23	-	-
10^{-3}	27.46	-	-
10^{-4}	31.03	-	-
10^{-5}	34.29	-	-
A/Indiana/27/2018(H3N2)v 2010.1			
neat	-	19.34	-
10^{-1}	-	22.21	-
10^{-2}	-	25.28	-
10^{-3}	-	27.95	-
10^{-4}	-	30.17	-
10^{-5}	-	32.54	-
A/Ohio/9/2015(H1N2)v 1A.3.3.3			
neat	-	-	15.36
10^{-1}	-	-	18.05
10^{-2}	-	-	21.55
10^{-3}	-	-	24.96
10^{-4}	-	-	28.04
10^{-5}	-	-	30.15

Table S3. Detailed results of IAV-S Triplex rRT-PCR assay and Next-Generation Sequencing from exhibition swine sampling

Pig	Ct value of rRT-PCR[1]			Subtype/Clade[2]	Pig	Ct value of rRT-PCR[1]			Subtype/lineage[2]
	sH1	H3v_2010	cH1			sH1	H3v_2010	cH1	
4	-	-	24.36		107	-	-	29.30	H1N1 /1A.3.3.3
5	-	-	27.65		108	-	-	25.13	H1N1 /1A.3.3.3
7	-	-	22.51		109	-	-	27.30	H1N1 /1A.3.3.3
13	-	-	28.53		110	-	-	26.62	H1N1 /1A.3.3.3
17	-	-	28.26		111	-	-	-	
26	-	-	-		112	-	-	29.29	H1N1 /1A.3.3.3
30	-	-	23.36	H1N1 /1A.3.3.3	113	-	-	-	
31	-	-	-		114	-	-	27.08	H1N1 /1A.3.3.3
35	-	-	-		115	-	-	-	
38	23.44	-	-	H1N2 /1B.2.1	116	-	-	-	
42	-	-	-		117	-	-	31.42	
44	22.70	-	-	H1N2 /1B.2.1	118	-	-	-	
45	-	-	27.40		119	-	-	-	
53	-	-	-	H1N1pdm09/1A.3.3.2	120	-	-	-	
55	-	-	-	H1N1pdm09/1A.3.3.2	121	-	-	-	
59	26.00	-	-	H1N2/ 1B.2.1	122	-	-	31.40	H1N1 /1A.3.3.3
65	-	-	27.30	H1N1 /1A.3.3.3	123	-	-	-	
79	-	-	20.88	H1N1 /1A.3.3.3	124	-	-	-	
90	-	-	-		125	-	-	-	H1N2 /1B.2-Other Human
94	31.75	-	-		126	-	-	-	
96	-	-	-		127	-	-	-	
97	-	-	-		128*	-	-	-	
98	24.99	-	-	H1N2/1B.2.1	129	-	-	-	
99	-	-	-		130	-	-	-	H1N1pdm09/1A.3.3.2
101	-	-	-		131	27.1	-	-	H1N2 1B.2.1
102	-	-	-		132	-	-	-	
103	-	-	-		133	-	-	-	H1N1pdm09/1A.3.3.2
104	-	-	-		134	-	-	-	
105	-	-	-		135	-	-	-	
106	-	-	29.02	H1N1 /1A.3.3.3	136	-	-	-	H1N1pdm09/1A.3.3.2

[1]Ct values from the IAV-S Triplex rRT-PCR for samples that were subsequently sequenced in-field.

[2]Swine influenza virus subtype and clade were determined from HA top Blast hit for the consensus sequence at 20x average coverage

*This sample did reach 20X coverage for HA gene but did not pass additional QC requirements, so was not included in the comparison of sequencing to swine influenza triplex rRT PCR assay results

Table S4. Assay limit of detection with swine influenza H1 and swine H3 2010.1 clade viruses with updated design.

**Swine Influenza A virus**	**Swine Triplex**				**FluSC2 Multiplex**			
A/Ohio/35/2017(H1N2)v 1B.2.1	cH1	H3v2010	sH1		InfA	InfB	SC2	RP
1.28x10^0.9	-	-	27.37/27.36/27.25		23.01/22.78/23.00	-	-	-
2.56x10^-0.1	-	-	28.60/28.96/28.82		24.43/24.90/24.62	-	-	-
5.12x10^-1.1	-	-	30.30/30.17/29.29		26.78/26.34/26.59	-	-	-
1.02x10^-1.1	-	-	30.89/31.46/31.41		28.22/27.73/27.83	-	-	-
2.05x10^-2.1	-	-	33.17/34.50/33.37		29.68/29.39/29.53	-	-	-
4.10x10^-3.1	-	-	35.97/ - /38.03		31.03/32.05/30.91	-	-	-
8.19x10^-4.1	-	-	- / - / 37.87		33.56/31.38/32.69	-	-	-
1.64x10^-4.1	-	-	- / - / -		34.57/35.60/36.19	-	-	-
A/Ohio/27/2016(H3N2)v 2010.1								
1.28x10^0.1	-	29.02/29.07/28.92	-		22.85/22.40/22.12	-	-	-
2.56x10^-0.9	-	30.98/31.00/31.02	-		25.15/25.19/25.30	-	-	-
5.12x10^-1.9	-	32.08/32.17/32.12	-		27.12/27.32/28.13	-	-	-
1.02x10^-1.9	-	33.16/33.14/33.14	-		29.89/29.99/29.89	-	-	-
2.05x10^-2.9	-	34.47/35.25/37.35	-		31.46/31.61/31.98	-	-	-
4.10x10^-3.9	-	- /38.43/36.58	-		34.18/34.57/34.03	-	-	-
8.19x10^-4.9	-	- / - / -	-		34.73/ - / -	-	-	-
1.64x10^-4.9	-	- / - / -	-		- / - /35.482	-	-	-
A/Ohio/24/2017(H1N2)v 1A.1.1								
1.28x10^0.3	29.28/29.15/29.15	-	-		22.25/22.49/22.58	-	-	-
2.56x10^-0.7	31.86/31.41/31.88	-	-		26.69/27.02/26.81	-	-	-
5.12x10^-1.7	32.75/32.86/32.96	-	-		28.85/29.37/28.65	-	-	-
1.02x10^-1.7	34.53/34.11/34.05	-	-		31.36/30.45/31.12	-	-	-
2.05x10^-2.7	35.84/36.84/38.65	-	-		33.05/34.93/34.31	-	-	-
4.10x10^-3.7	39.57/37.58/37.69	-	-		- /35.71/35.65	-	-	-
8.19x10^-4.7	39.79/ - / -	-	-		35.37/ - / -	-	-	-
1.64x10^-4.7	- / - / -	-	-		- / - / -	-	-	-
